# GWYRE: A Resource for Mapping Variants onto Experimental and Modeled Structures of Human Protein Complexes

**DOI:** 10.1016/j.jmb.2022.167608

**Published:** 2022-06-15

**Authors:** Sukhaswami Malladi, Harold R. Powell, Alessia David, Suhail A. Islam, Matthew M. Copeland, Petras J. Kundrotas, Michael J.E. Sternberg, Ilya A. Vakser

**Affiliations:** 1 Computational Biology Program, The University of Kansas, Lawrence, KS 66047, USA; 2 Centre for Integrative Systems Biology and Bioinformatics, Department of Life Sciences, Imperial College London, South Kensington, London SW7 2AZ, UK; 3 Department of Molecular Biosciences, The University of Kansas, Lawrence, KS 66045, USA

**Keywords:** structure prediction, protein docking, genotype to phenotype, amino acid mutations, genome-wide modeling

## Abstract

Rapid progress in structural modeling of proteins and their interactions is powered by advances in knowledge-based methodologies along with better understanding of physical principles of protein structure and function. The pool of structural data for modeling of proteins and protein–protein complexes is constantly increasing due to the rapid growth of protein interaction databases and Protein Data Bank. The GWYRE (Genome Wide PhYRE) project capitalizes on these developments by advancing and applying new powerful modeling methodologies to structural modeling of protein–protein interactions and genetic variation. The methods integrate knowledge-based tertiary structure prediction using Phyre2 and quaternary structure prediction using template-based docking by a full-structure alignment protocol to generate models for binary complexes. The predictions are incorporated in a comprehensive public resource for structural characterization of the human interactome and the location of human genetic variants. The GWYRE resource facilitates better understanding of principles of protein interaction and structure/function relationships. The resource is available at http://www.gwyre.org.

## Highlights

• Structure of protein complexes is important for interpreting genetic variation.
• Data on single amino acid variants is available from high-throughput sequencing.
• Integrated modeling approach was applied to proteins and their complexes.
• GWYRE resource incorporates predicted protein complexes with mapped mutations.

## Graphical abstract

**Figure:**
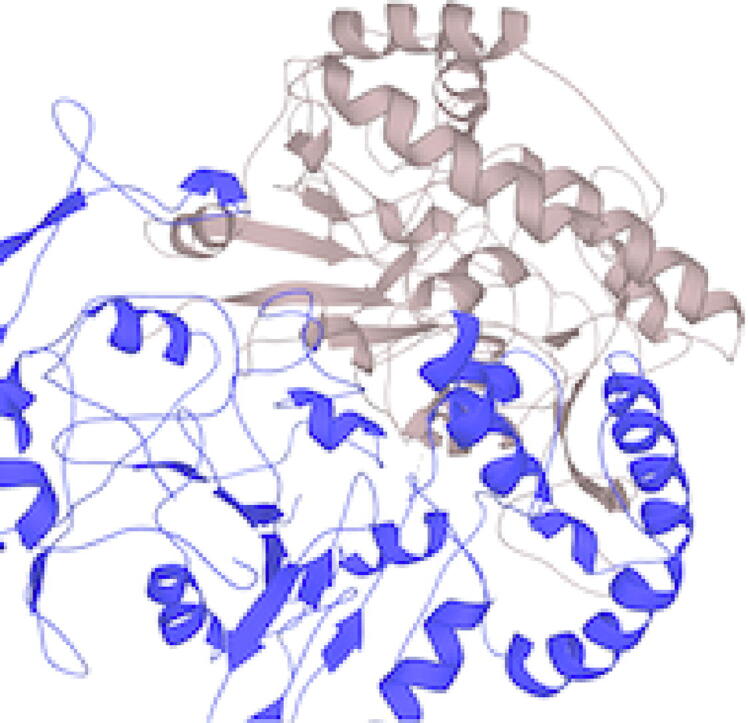

## Introduction

Structural characterization of protein interactome^1^ is essential for interpretation of genetic variation.^2^, ^3^ A vast amount of information on human genetic variation, including numerous single amino acid changes, is available from high-throughput sequencing. Despite significant progress in experimental techniques for protein structure determination, which fuels remarkable expansion of the Protein Data Bank (PDB),^4^, ^5^ structures of most proteins must be determined by modeling. The number of protein–protein interactions (PPI) is significantly larger than the number of individual proteins. Moreover, structures of protein assemblies are more difficult to determine experimentally than that of the individual proteins, which makes the role of modeling in structural characterization of the interactome even more important.^6^, ^7^, ^8^, ^9^

Computational approaches to structure determination of individual proteins and protein–protein complexes have been rapidly progressing.^10^ Development of approaches based on deep learning, in particular by AlphaFold,^11^ opens a new chapter in the structure prediction field. However, in less challenging, high-throughput applications, when coarse-grained predictions suffice for further analysis, less demanding, faster approaches (such as template-based modeling) are still valid.^12^

There are several databases that report human protein–protein interactions (e.g., IntAct,^13^ BioGRID^14^ and STRING^15^ ), with BioGRID and STRING reporting protein–protein interactions in several other organisms. UniProt^16^ provides a single resource reporting human genetic variation combining data from 100 K genomes, ExAC, ClinVar, TCGA, COSMIC, TOPMed and gnomAD. The interpretation of how these genetic variants impact protein interactions greatly benefits from structural models that can be examined and analyzed. Accordingly, several groups have provided resources that map the location of genetic variants reported in databases onto protein structure. Several resources just consider experimental structures such as PDBe-KB^17^ and ADDRESS.^18^ Other resources include both experimental structures (including multi-chain, as available in the PDB) and modeled tertiary structures such as PhyreRisk,^19^ DeepSAV^20^ and MSV3d.^21^ The extent of structural coverage can be enhanced by predicting quaternary structure in addition to the tertiary structure. Interactome3D^22^ contains experimental interaction structures as well as docking models generated using sequence-based template search. Extending Interactome3D, the team have developed the dSysMap database which maps genetic variants onto both experimental and predicted structures including binary complexes.^23^ Docked structures in dSysMap are predicted based on templates of experimental complexes, again found by the sequence homology.

We report the GWYRE (Genome Wide PhYRE) resource, which currently integrates knowledge-based tertiary structure prediction using Phyre2^24^ and quaternary structure prediction using template-based docking by full-structure alignment.^25^ The search for the docking template is based on the structure similarity rather than sequence similarity, which leads to significant expansion of the templates pool.^26^ The predictions are incorporated in a comprehensive web-based public resource for structural characterization of interactomes and mapping of missense variants obtained from UniProt. The resource, available at https://www.gwyre.org, facilitates better understanding of principles of protein interaction and structure/function relationships. Coordinates of complexes can be downloaded for inspection and further analysis.

## Results and Discussion

### GWYRE overview

The GWYRE database provides mapping of human coding variations onto experimental and modeled protein structure and complexes, thus providing a valuable resource for the scientific community engaged in understanding how genetic variants affect phenotype.

The GWYRE database contains (as of November 29, 2021; more structures are being currently processed):

1. 2,797 experimentally determined entries (X-ray and cryoEM, obtained from the PDB and presented “as is”. For these entries, data on 363,836 mutations for 876 unique (by UniProt ID) proteins was downloaded from UniProt on August 25, 2021.
2. 907 “PDB + PDB” entries generated by docking two experimental structures (obtained from PDB). For these entries, data on 292,404 mutations for 646 unique proteins was downloaded from UniProt on October 11, 2021.
3. 586 “PDB + model” entries obtained by docking the PDB structure of one interactor and a 3D model of the other protein. For these entries, data on 226,624 mutations for 658 unique proteins was downloaded from UniProt on November 8, 2021.
4. 2,351 “model + model” entries obtained by docking two 3D models of the interacting proteins. For these entries, data on 366,181 mutations for 1352 unique proteins was downloaded from UniProt on September 1, 2021.

**Figure 1:**
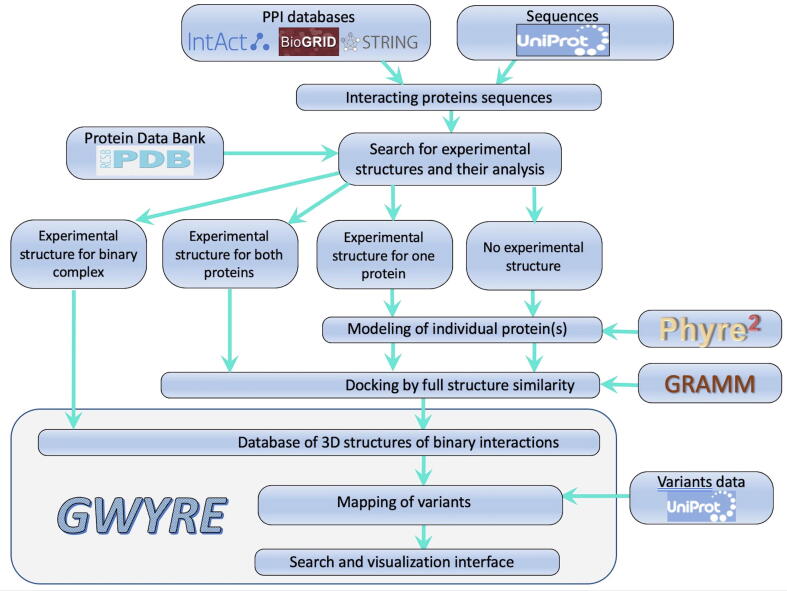
Modeling pipeline.

In total, GWYRE provides structures for 6641 complexes onto which the location of 1,249,045 mutations is mapped. The overview of the GWYRE operational sequence is in Figure 1.

### Import and analysis of protein interaction data

All binary protein–protein interactions with both proteins from human (by taxonomy ID 9606) were imported from IntAct,^13^ BioGRID^14^ and STRING^15^ (physical interactions only) databases containing 580,375 PPI at the time of the download (May 2021). For this study, we kept only PPI where both protein sequences could be mapped to canonical UniProt sequence (568,486 PPI involving 18,423 proteins). By searching sequences from PDB, we identified 2,797 PPI, for which an experimental structure was available (“experimental structures” GWYRE entries). For the NMR structures, we used the first model. In the case of homo-dimeric interactions, experimental structures were retained only if the homodimer was present in the biological unit of the PDB entry. If the homo-oligomeric state in the biounit was >2, we chose the interface with the largest interface area. We also identified 27,770 PPI, for which an experimental structure was available for both interactors in different PDB entries (“PDB + PDB” GWYRE entries), and 44,488 PPI, for which a PDB structure was available for one of the interactors (“PDB + model” GWYRE entries). For all PDB entries in GWYRE, we required that the experimental structure covers at least 80% of the protein UniProt sequence. In the case of multiple PDB structures with such coverage, we choose the representative structure with the largest coverage, the smallest number of missing atoms/residues, the experimental method (X-ray first, then cryo-EM, then NMR), the best resolution and/or the latest deposition date. All sequences without such a PDB structure (15,272 in total) were submitted to the Phyre2 modeling pipeline. All the 2,797 experimental complexes are in GWYRE with the remaining sequences and structures being processed as below (only those passing our restrictive quality checks being included in GWYRE).

### Modeling of individual proteins

The aim was to use our Phyre2 homology modeling server^24^ to predict the structure of proteins prior to the docking. The requirement was to generate models for the entire protein chain rather than partial structures which lack substantial regions, including one or more domains, as these predictions were then going to be docked into a complex and partial structures could lead to generating false docking poses. Our trials showed that for sequences of >500 residues, Phyre2 was only able to generate very few full-length quality models (see below for definition of quality). Accordingly, each sequence (identified by its UniProt Accession) with ≤500 residues was submitted to the Phyre2 server for homology modeling.

Phyre2 was run in “normal mode” where a single PDB structure provides the template. As NMR structures provide an ensemble of structures, these were not selected as a template. Insertions and deletions were modeled by identifying PDB fragments that can be melded onto the fixed regions. Side chains were then added and the optimum packing of rotamers established as reported.^24^

Phyre2 generates a ranked list of hits based on increasing E-values from the HHSearch.^27^ The following criteria were applied to exclude poor quality solutions:

• ≥90% Confidence (i.e., “Probability”) from HHSearch.
• The template used for Phyre2 had >20% sequence identity with the target sequence as defined by HHSearch.
• No missing segments in the model of >30 consecutive residues either within the sequence or at the N- or C-termini.
• No unreasonably large distance between the C^α^ atoms of consecutive residues. A value of 3.8 Å x gap length in residue number + 1.2 Å was used.
• To avoid elongated or severely flattened molecular envelopes, which may present difficulties in docking, a predicted structure had to meet the following two tests on its shape: (*i*) radius of gyration < 0.8, i.e., the RMS distance of the center of mass of an object from its axis of rotation. It can be taken as a measure of the deviation from *mmm* symmetry, e.g., banana shaped as opposed to ellipsoidal; and (*ii*) the anisotropy of the principal component analysis (PCA) is <4.0; PCA is used to determine the ellipticity of a distribution. A spherical distribution has an anisotropy of unity, while prolate or oblate spheroids have larger values.

Phyre2 produces a list of solutions, of which the best 20 were modeled, where the ranking is based on the E-value from HHSearch. The top hit that met the above criteria was selected except for two situations. The first situation is if there was a lower ranking Phyre2 hit derived from a human protein corresponding to the query UniProt sequence in the top 20 hits. This was selected provided the coordinates were obtained from either (i) a single-crystal diffraction (X-ray, electron, or neutron) method or (*ii*) single particle cryo-electron microscopy. For most sequence queries, the top hit actually corresponded to the human template. The second situation arises when the Phyre2 template library only contains representative domains where no two entries have >70% sequence identity. Thus, there could be a structure of a human protein available in the PDB but not in the template library. Accordingly, where the Phyre2 template library did not contain an entry corresponding to a human protein, but an entry existed in the PDB, Phyre2 was run in the “one-to-one threading mode”, where the sequence of the protein from the UniProt entry is aligned against that from the individual PDB entry rather than against the entire fold library. The motivation for running Phyre2 when there is an available PDB structure for that sequence is that often the PDB entry can have missing atoms, and these would be modeled without introducing substantive conformational changes to the remainder of the protein where coordinates are available.

A breakthrough in the modeling of tertiary structures occurred with the release of the second generation of the AlphaFold software.^28^ The AlphaFold pipeline consists of several deep neural networks with sophisticated architectures (self-attention, convolution, transformers, transfer learning, etc.), which essentially establish connection between 2D residue-residue distances (contact maps) and 3D arrangements of atoms of those residues (in spirit, similar to the NMR technique). Since the AlphaFold was released after the main body of modeling work in this study had been accomplished, we did not incorporate AlphaFold-based models in the current GWYRE version, but plan to do this in the future GWYRE releases. To incorporate AlphaFold predictions, one would need to develop an approach to identify when the relative position of protein domains is accurate.^12^

### Protein-protein docking

Most newly released PDB structures of protein–protein complexes have easily identifiable homologs among previously determined structures, which could have been used as templates for their modeling (Koirala et al. unpublished results). Thus, template-based approaches to protein docking provide a viable solution to structural characterization of many protein–protein complexes. The template-based docking was performed on PDB structures (1,792 chains) and modeled structures (3,598 chains) of individual proteins by the full structure alignment protocol,^25^ using our most recent template library of 11,756 co-crystallized binary complexes from Dockground.^29^ The target proteins were structurally aligned to the template monomers by TM-align.^30^ Only alignments with target/template TM-scores^31^ > 0.4 were used to build the docking models further scored by the combined scoring function.^32^ In this GWYRE release, we kept only docking models with this score > 0.5 as benchmarking studies^32^ showed that 99 % of models with such score are of acceptable or better quality according to the CAPRI criteria. We did not perform any refinement of the resulting model as our study^33^ showed that the near-native docking models generated by the above approach do not have a significant number of clashes at the interface. This protocol resulted in 907 “PDB + PDB”, 586 “PDB + model” and 2,351 “model + model” docked complexes (as of November 29, 2021). The distribution of target/template sequence identities for the models of individual proteins (1263 chains) in the final docking models in the current GWYRE release is shown in Supplementary Figure S1. This is directly related to the accuracy of individual protein models as was reported previously^34^ (for 90–95% the median root mean square deviation of superposed C_α_ atoms is 0.86 Å and for 30–39% it is 2.79 Å).

In the future GWYRE development, we plan to extend pool of the docking models by including models generated by the partial structural alignment and free docking by GRAMM^35^, ^36^ and, when applicable, AlphaFold-multimer.^37^

### User interface

**Figure 2:**
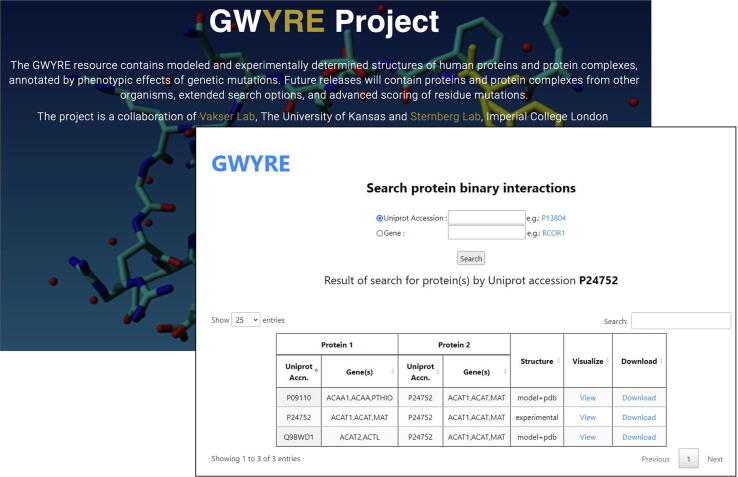
GWYRE home page and an example of the search page.

The GWYRE resource is available at https://gwyre.org (Figure 2). The home page contains the project background and links to the download and search of the docked complexes in PDB format. The search can be performed by either the gene or the protein name. The search output is a list of interacting proteins, the type of structure (experimentally determined or modeled) and links to the visualization of the docked structure along with the variants, and to the download of PDB-formatted file of the docked structure.

**Figure 3:**
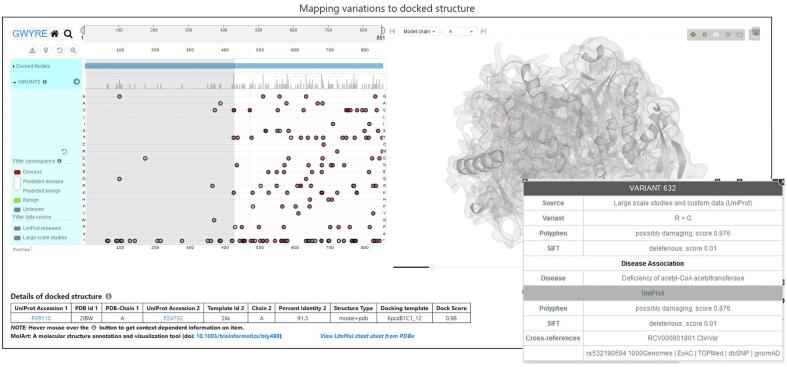
Example of the visualization page and popup window for the variant 632 in the docked structure (residue 207 in the protein P24752).

The visualization page (Figure 3) utilizes the ProtVista^38^ interface which allows viewing variants mapped onto the sequence of the protein. Mapping was performed by aligning protein sequences extracted from ATOM section of PDB file and corresponding concatenated UNIPROT sequences. Sequence positions can be zoomed in and panned to narrow down the regions of interest. These regions are highlighted on the 3D docked structure, visualized using LiteMol viewer.^39^ Mapping of the protein sequence features onto the docked structure is performed by the MolArt JavaScript plugin.^40^ Variations on the ProtVista interface are shown as circles (one circle per variant) aligned on the 1D sequence representation. Colors of the circles correspond to four types of the variants: associated with disease (red, at least one experimental study pointing to a specific disease associated with that variant), benign (green, all experimental studies do not point to any disease associated with that variant), predicted consequences (different shades of blue depending on the prediction score, from Polyphen^41^ and/or sometimes SIFT,^42^ ranging from dark blue, disease, to light blue, benign), and unknown (gray, no experimental studies or predictions). Variants can be shown separately for each variant type and filtered by the data source (currently, we included reviewed Uniprot entries and large-scale studies) by clicking on appropriate colored or gray boxes. Hovering mouse over a circle shows the wild-type and the variant residues along with the source from which the variant was obtained. The corresponding part of the 3D structure is also highlighted. More information on the items listed on the screen can be obtained by hovering the mouse over on the ‘*i*’ and ‘?’ buttons next to the ProtVista and LiteMol items, respectively. The table at the bottom of the screen shows the details of the binary docking including UniProt accessions of the individual proteins, PDB name and chains of the experimentally determined protein structures or the modeling template for the Phyre2 modeled structures, the type of the docked structure (e.g., “model + model”, “model + PDB”, etc.), as well as sequence identities for the individual models (if applicable), docking template and the overall docking score.

### Resource content and implementation

The GWYRE resource consists of PDB formatted files, each containing two docked proteins. For consistency, proteins are labeled ‘A’ and ‘B’ for the larger and the smaller protein (based on the lengths of canonical UniProt sequences) in the pair, respectively. The chain IDs may differ from those in the original PDB file. Residues in the GWYRE PDB-formatted files are renumbered to correspond to the numbering in the full canonical UniProt sequence. This ensures correct structural mapping of the variants. Sequences, features of the individual proteins and interaction details are stored in a PostgreSQL relational database, which is queried using SQL statements. The web page is written in PHP and JavaScript. Processing of the data before and after docking is performed by R scripts.

### Example

Figure 2 shows search results for protein P24752. The protein (mitochondrial Acetyl-CoA acetyltransferase) is one of the enzymes that catalyzes the last step of the mitochondrial beta-oxidation pathway, an aerobic process breaking down fatty acids into acetyl-CoA.^43^, ^44^, ^45^ Its canonical sequence consists of 427 amino acids in 2 PFAM domains: Thiolase N (residues 42–299) and Thiolase C (residues 306–426). The protein was crystallized as a homo-tetramer (in both biological and asymmetric PDB units) in seven PDB entries. According to our criteria, PDB 2ibw was selected as representative. This protein participates in 180 interactions with other human proteins, which can be mapped to the canonical UniProt sequence. However, currently GWYRE, due to strict requirements on the quality of individual and docked models, contains data only for 3 PPI (shown in Figure 3). One PPI is the experimental structure of a homodimer, consisting of chains C and D of 2ibw. The other two are complexes of docked chain A of 2ibw and the high-quality Phyre2 models for proteins Q9BWD1 and P09110, produced by Phyre2 by using chain A of 1wl5 and chain A of 2iik respectively. Figure 3 shows the mapping/visualization screen for the PPI of P24752 and P09110 (424 resides peroxisomal 3-ketoacyl-CoA thiolase). UniProt reported, in total, 829 variants for this PPI (all 100 + predicted mutations were removed for clarity). All 123 disease-associated variants are present only for one of the proteins, P24752, while 2 out of 3 benign variants are observed for another protein. There are 23 and 16 variants of unknown consequence for the first and the second protein, respectively and 734 predicted variants uniformly distributed between both proteins. Out of those predictions, 54% have Polyphen score > 0.5 (likely disease causing) and the rest can be viewed as likely benign. When pointing the mouse over a mutation, a popup shows the details of that mutation and highlights the position of that residue in the 3D structure. This docking structure is of “model + pdb” type, thus table at the bottom provides information on Uniprot Accession numbers, information on the experimental structure of the first protein (PDB code in capital letters and chain ID), template details for the PHYRE2 model of the second protein (PDB code in small letters, chain ID and sequence identity), docking template and the score for the displayed structure of the complex.

## Conclusions

Rapid progress in structural modeling of proteins and their interactions is powered by advances in knowledge-based methodologies along with better understanding of physical principles of protein structure and function. The pool of structural data for modeling of proteins and protein–protein complexes is constantly increasing due to the rapid growth of protein interaction databases and PDB. The GWYRE project capitalizes on these developments by advancing and applying new powerful modeling methodologies to structural modeling of protein–protein interactions and single amino acid variation. The methods integrate knowledge-based tertiary structure prediction using Phyre2 and quaternary structure prediction using template-based docking by GRAMM. The predictions are incorporated in a comprehensive public resource for structural characterization of interactomes and assessment of phenotypic effects of genetic variation. The utility to download coordinates of both experimental and predicted binary complexes of interacting human proteins from GWYRE facilitates further analysis including computational assessment of the effect of missense variants using approaches such as FoldX,^46^ mCSM^47^ and BeAtMuSIC.^48^ To conclude, the GWYRE resource, available at https://www.gwyre.org, facilitates better understanding of principles of protein interaction and structure/function relationships.

### CRediT authorship contribution statement

**Sukhaswami Malladi:** Software, Data curation, Validation, Visualization, Writing – original draft. **Harold R. Powell:** Software, Data curation, Validation, Writing – original draft. **Alessia David:** Methodology, Investigation, Data curation, Writing – original draft. **Suhail A. Islam:** Software, Data curation, Resources. **Matthew M. Copeland:** Software, Data curation, Visualization, Resources. **Petras J. Kundrotas:** Funding acquisition, Project administration, Supervision, Methodology, Writing – review & editing. **Michael J.E. Sternberg:** Funding acquisition, Conceptualization, Project administration, Supervision, Writing – review & editing. **Ilya A. Vakser:** Funding acquisition, Conceptualization, Project administration, Supervision, Writing – review & editing.
