# Supplementary material for: GWYRE: A Resource for Mapping Variants onto Experimental and Modeled Structures of Human Protein Complexes
Source: J Mol Biol. Author manuscript; Available in PMC 2022 Jul 29. (PMC9188266; doi:10.1016/j.jmb.2022.167608)
Supplement: Supplementart file [file EMS145852-supplement-Supplementart_file.pdf]

Supplemental Materials

GWYRE: A resource for mapping variants onto experimental and modeled structures of human protein complexes

Sukhaswami Malladi, Harold R. Powell, Alessia David, Suhail A. Islam, Matthew M. Copeland, Petras J. Kundrotas, Michael J.E. Sternberg, Ilya A. Vakser

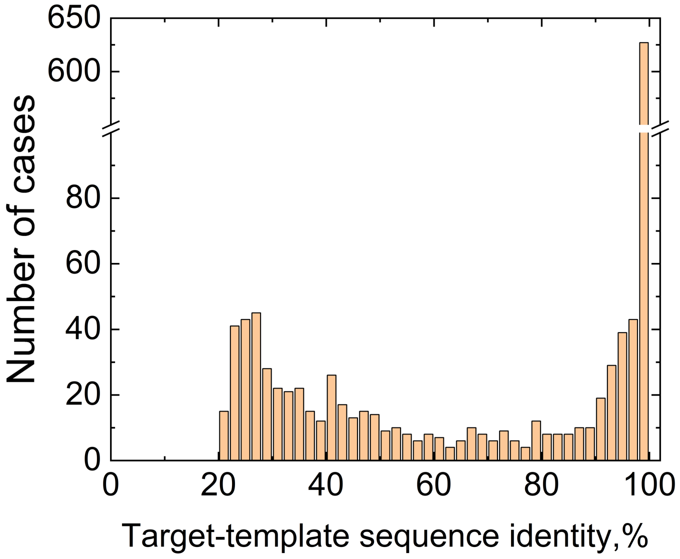

Figure S1. Distribution of target-template sequence identities in the final models of individual proteins produced by Phyre2 in the current GWYRE release.
